# Deep Learning for Identification of Alcohol-Related Content on Social Media (Reddit and Twitter): Exploratory Analysis of Alcohol-Related Outcomes

**DOI:** 10.2196/27314

**Published:** 2021-09-15

**Authors:** Benjamin Joseph Ricard, Saeed Hassanpour

**Affiliations:** 1 Department of Biomedical Data Science Dartmouth College Lebanon, NH United States; 2 Department of Epidemiology Dartmouth College Hanover, NH United States; 3 Department of Computer Science Dartmouth College Hanover, NH United States

**Keywords:** social media, natural language processing, alcohol abuse, machine learning

## Abstract

**Background:**

Many social media studies have explored the ability of thematic structures, such as hashtags and subreddits, to identify information related to a wide variety of mental health disorders. However, studies and models trained on specific themed communities are often difficult to apply to different social media platforms and related outcomes. A deep learning framework using thematic structures from Reddit and Twitter can have distinct advantages for studying alcohol abuse, particularly among the youth in the United States.

**Objective:**

This study proposes a new deep learning pipeline that uses thematic structures to identify alcohol-related content across different platforms. We apply our method on Twitter to determine the association of the prevalence of alcohol-related tweets with alcohol-related outcomes reported from the National Institute of Alcoholism and Alcohol Abuse, Centers for Disease Control Behavioral Risk Factor Surveillance System, county health rankings, and the National Industry Classification System.

**Methods:**

The Bidirectional Encoder Representations From Transformers neural network learned to classify 1,302,524 Reddit posts as either alcohol-related or control subreddits. The trained model identified 24 alcohol-related hashtags from an unlabeled data set of 843,769 random tweets. Querying alcohol-related hashtags identified 25,558,846 alcohol-related tweets, including 790,544 location-specific (geotagged) tweets. We calculated the correlation between the prevalence of alcohol-related tweets and alcohol-related outcomes, controlling for confounding effects of age, sex, income, education, and self-reported race, as recorded by the 2013-2018 American Community Survey.

**Results:**

Significant associations were observed: between alcohol-hashtagged tweets and alcohol consumption (*P*=.01) and heavy drinking (*P*=.005) but not binge drinking (*P*=.37), self-reported at the metropolitan-micropolitan statistical area level; between alcohol-hashtagged tweets and self-reported excessive drinking behavior (*P*=.03) but not motor vehicle fatalities involving alcohol (*P*=.21); between alcohol-hashtagged tweets and the number of breweries (*P*<.001), wineries (*P*<.001), and beer, wine, and liquor stores (*P*<.001) but not drinking places (*P*=.23), per capita at the US county and county-equivalent level; and between alcohol-hashtagged tweets and all gallons of ethanol consumed (*P*<.001), as well as ethanol consumed from wine (*P*<.001) and liquor (*P*=.01) sources but not beer (*P*=.63), at the US state level.

**Conclusions:**

Here, we present a novel natural language processing pipeline developed using Reddit’s alcohol-related subreddits that identify highly specific alcohol-related Twitter hashtags. The prevalence of identified hashtags contains interpretable information about alcohol consumption at both coarse (eg, US state) and fine-grained (eg, metropolitan-micropolitan statistical area level and county) geographical designations. This approach can expand research and deep learning interventions on alcohol abuse and other behavioral health outcomes.

## Introduction

### Background

Alcohol-related causes are the third leading preventable cause of death in the United States, and alcohol abuse contributes to many adverse health outcomes, particularly on the developing brain [1-4]. The rise of alcohol-related content on Twitter is alarming, with over half of young adults participating in a study [5] posting alcohol-related content. Social media use and alcohol consumption are common behaviors; the prevalence rates of Twitter, Reddit, and annual alcohol use for US adults are 22%, 11%, and 70%, respectively [6,7]. Internet- and social media–based interventions are scalable and efficient approaches for developing practical tools for treating and monitoring alcohol abuse, especially for at-risk adolescents and young adults [8-14]. However, identifying high-risk areas for efficient and helpful monitoring along with population-level interventions remains a difficult task, in part because of survey bias [15-17].

Text-based *hashtags* are common among many popular social media platforms such as Twitter, Instagram, and TikTok. Individuals use hashtags to categorize, label, organize, and discover posts and content [18]. Previous studies have indicated that study-specific hashtags are useful for mental health research [19]. For example, sexual abuse and harassment (#MeToo), breast cancer (#breastcancer), HIV (#HIV), miscarriages (#ihadamiscarriage), tobacco use (#Vapelife), and viral pandemics (#COVID-19) are some of the many important health outcomes that have been previously studied using hashtags on Twitter [20-28]. Other social media platforms such as Reddit contain specific *themed* communities where interested users discuss a particular topic. In contrast to hashtags, themed communities on websites such as Reddit represent posts related to exactly 1 topic of interest. Like hashtags, these communities, such as *r/cripplingalcoholism*, *r/depression*, or *r/opiates* Reddit subreddits and *HIV* Baidu Tieba bar, contain information that can target and understand behavioral health and disease [29-33]. In addition to hashtags and subreddits, some social media platforms allow for *geotagging* or sharing a user's geographical latitude and longitude coordinates in a post. Geotags have been used in social media research to identify geographically relevant information from social media data [34-36].

### Previous Work

Although prior studies have identified specific hashtags or themed communities for studying behavioral health outcomes, many insights are platform-specific. Although helpful information regarding a behavior of interest or themed community may be available on one platform, there may not be such knowledge available on a different platform. Many previous methods examining alcohol content on social media use data from a single platform [5,37-42]. Single-platform analyses may limit discoveries and interventions to only a fraction of the population at risk. There is a growing need for behavioral health researchers working with social media data to incorporate analyses from many sources [43,44]. Although some studies have examined alcohol content on multiple platforms, many methods need survey data from known active users from each source or additional manual annotation [45-47]. The ability and insights gained from using deep learning methods to learn from a large number of posts from specific communities (ie, Reddit subreddits) to predict alcohol-related content on a different platform (ie, Twitter) remain unclear.

Many previous studies that identified alcohol-related language on social media platforms relied on training on extrinsic labels, such as survey responses. Reliance on self-report data is problematic as alcohol consumption is subject to bias, particularly among the youth [15,16,48]. In addition, approaches that use an outcome of interest to both train and evaluate a model (eg, identifying and evaluating alcohol-related hashtags or keywords based on enrichment in regions with higher self-reported alcohol content) may not be generalizable to other related outcomes [49].

Other approaches for studying alcohol content involve identifying a sample as being alcohol-related based on the identification of keywords. Keyword approaches have distinct benefits, such as interpretability. However, identifying text from keywords may rely on standard and predefined terms (eg, searching *drunk*), training on self-report data, or manual review [37,42,49-51]. Classification of social media posts based on previously defined keywords or vector representations (eg, Word2vec) is not as useful when the average length of sequences is small and has out-of-training vocabulary [52-54]. Training on nonspecific platform information alone may fail to capture relevant keywords, especially for rarer outcomes not prominent in the heterogeneity of random and unlabeled social media chatter [55]. In addition, predefined keywords or word vectors may fail to capture slang or the different language structures between Reddit and Twitter [56].

One recent contribution in natural language processing (NLP) is the Bidirectional Encoder Representations From Transformers (BERT) neural network, which has demonstrated superior performance on a wide variety of social media NLP tasks [57-61]. BERT focuses on learning by analyzing sentences with randomly masked words. This masked language model deconstructs larger strings into smaller tokens and is ideal for dealing with hashtags and other platform-unique token structures [57]. Before developing BERT, previous models, such as long-short–term memory networks, logistic regression, Word2vec similarity, and latent Dirichlet allocation, were not well suited to process unknown words and hashtag structures. For example, some previous NLP studies on social media either removed hashtags, represented them as universal tokens, or removed *#* from strings, with no importance given to hashtags (eg, *#ilovebeer* represented as “ ” (space), *HASHTAG*, or *ilovebeer*, respectively) [62-64]. In contrast, using hashtags and themed communities as explicit labels in a deep learning architecture allows for identifying relevant, platform-specific hashtags that can identify posts that indicate the behavior of interest. In addition, the use of these structures adds a layer of interpretability to our trained neural networks, which are commonly criticized as noninterpretable black boxes [65].

Other previous social media text mining methods implementing deep learning often involve training platform-specific models. One issue with this approach is that each platform’s training models require an extensive amount of usually labeled data from that platform [66-72]. In addition, although deep learning models have been successful at many tasks, training platform-specific deep networks such as BERT (containing >100 million parameters) is extremely energy- and cost-intensive, and CO_2_ emissions from training BERT models have raised concerns about their environmental impact [73]. Optimal methods for translating information from previously trained social media deep learning models to discern insights from separate social media platforms remain a relatively unexplored research area.

### The Goal of This Study

We aim to examine the effectiveness of using thematic structures in a deep learning framework to identify alcohol-related behaviors across different social media platforms. First, we trained on Reddit subreddits to identify alcohol-related targets on another social media platform (Twitter) with a different thematic structure (hashtags). Next, we determined whether the hashtags predicted by the model correlate to known alcohol-related outcomes, including self-reported drinking status, alcohol outlet density, and estimated gallons of ethanol consumed, after controlling for confounding effects of age, sex, income, education, and self-reported race. We show that these data-driven hashtags contain interpretable information about alcohol consumption in the United States. Finally, we present validated and queryable hashtags from our model that behavioral health researchers can use as a starting point for the identification of alcohol-related content on Twitter, Reddit, and other social media platforms.

## Methods

### Overview of the NLP Pipeline

**Figure 1:**
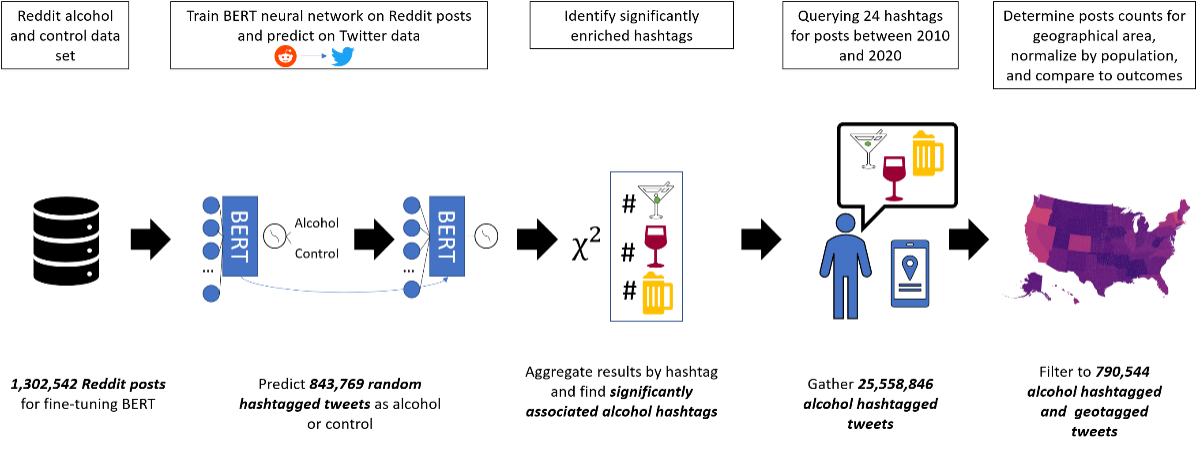
Overview of the methodological pipeline. A bidirectional encoder representation from transformers model trained to classify posts as either 18 alcohol-related or control subreddits. The bidirectional encoder representations from transformers model was applied to a set of tweets containing at least one hashtag. The prediction results were analyzed to find 24 significantly enriched hashtags as positive predictions (ie, prediction probability ≥0.5). Tweets posted between 2010 and 2020 with an alcohol-related hashtag were collected and filtered on geotagged location. BERT: Bidirectional Encoder Representations From Transformers.

**Figure 2:**
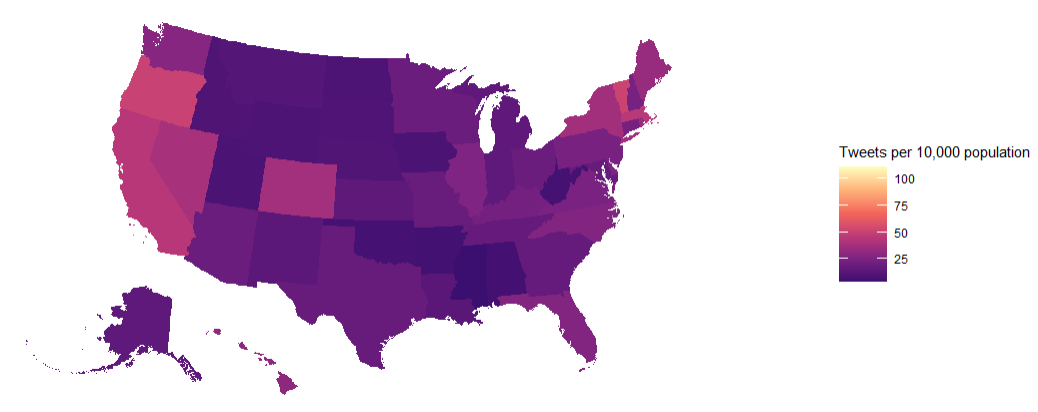
Choropleth of the US state and Washington, DC tweets with alcohol-related hashtags per 10,000 persons.

This study fine-tuned a BERT neural network as a binary classifier to predict Reddit post titles as belonging to either alcohol-related communities or a random subreddit. Next, we applied the Reddit-trained network to a smaller set of random, unlabeled Twitter posts to identify 24 hashtags that were significantly associated with alcohol content. We identified 25,558,846 tweets that contained at least one alcohol-related hashtag for the period between 2010 and 2019. A total of 1,412,041 alcohol-related tweets included latitude and longitude data from *geotagging*. The locations of 790,544 geotagged tweets from 2929 US counties and county equivalents were identified using data from the 2017 US Census Shapefiles database [74,75]. Finally, we examined the relationship between the prevalence of alcohol-related tweets per population and various outcome measures related to alcohol consumption, including self-reported alcohol consumption and alcohol outlet density. Figure 1 demonstrates an overview of our NLP pipeline. Figure 2 illustrates the choropleth of population-normalized alcohol-hashtagged tweets for US states and Washington, DC.

### Reddit Data Set and BERT Training

A large amount of alcohol-related training data were extracted from Reddit subreddits with the pushshift application programming interface (API) previously used in social media research [63,76]. On a subreddit, community moderators create description texts that contain links to other, usually related, subreddits. Scraping the description pages for all subreddits containing at least 1000 posts for any links to *r/drunk*, one of the most popular alcohol-related subreddits, and all links from *r/drunk* to other subreddits yielded 17 alcohol-related subreddits. A total of 651,271 post titles from the following 18 subreddits were used as positive alcohol labels for model training: *r/cripplingalcoholism*, *r/vodka*, *r/oldtimehockey*, *r/alcohol*, *r/beer*, *r/bourbon*, *r/homebrewing*, *r/drinkinggames*, *r/wine*, *r/beercirclejerk*, *r/gin*, *r/scotch*, *r/liquor*, *r/showerbeer*, *r/absinthe*, *r/firewater*, *r/beercanada*, and *r/drunk*.

Negative alcohol (control) posts were gathered by querying 651,271 random posts posted in all other subreddits, excluding the 18 alcohol-related subreddits. Training 79.99% (521,016/651,271), validation 9.99% (65,127/651,271), and testing 9.99% (65,127/651,271), data sets were generated for developing and evaluating the model—a binary classifier trained for posts belonging to either alcohol-related subreddits or other random subreddits. The model fine-tuned a pretrained BERT model with 12 layers and 768 hidden units in PyTorch on an NVIDIA TITAN Xp graphics processing unit using a batch size of 64 for approximately 5 weeks [77].

### Twitter Data Set and Identification of Hashtags

The Twitter API provides tweet information from 7 days before a query. Randomly selected tokens in the Twitter GLoVE word embedding dictionary and their respective hashtags (ie, a string that starts with *#*) were queried using the Twitter API to identify recently posted tweets containing that word or hashtag [78]. Each identified hashtag in the data set was requeried to ensure that it was monitored for at least 2 weeks. The initial *random* Twitter data set comprised 843,769 random hashtag-containing tweets posted between January 2019 and October 2019. The Reddit-trained BERT model was applied to this data set to obtain binary predictions for each tweet. A chi-square test identified 24 significant alcohol-related hashtags from posts predicted to be alcohol-positive (ie, final softmax layer prediction *P* value of ≥0.5) relative to posts predicted as negative (ie, final softmax prediction *P* value of <0.5) using a one-tailed *greater* test. We included only hashtags with 5 or more occurrences and applied the Benjamini-Hochberg algorithm for multiple hypothesis correction using a 0.05 false discovery rate, a common approach for multiple hypothesis corrections in social media data analyses [37,42,79-85]. The analysis resulted in 24 hashtags, as indicated in Textbox 1. GetOldTweets, a Python package widely used in social media research, was used to identify 25,558,846 alcohol-hashtagged tweets posted throughout 10 years (between 2010 and 2020) containing at least one significant alcohol-related hashtag [55,86,87].

**Alcohol-related hashtags extracted by our Reddit-trained classifier according to alcohol category.**

**Beer hashtags**

- *craftbeer*, *beer*, *ncbeer*, *brewery*, *stout*, *beeroclock*, *beergeek*, *beerporn*, *beers*, *instabeer*, *beertime*, *beerstagram*, *beerlover*, *beersnob*

**Wine hashtags**

- *winetasting*, *wine*, *winelover*, *wines*, *redwine*

**Liquor hashtags**

- *bourbon*, *whiskey*, *whisky*

**Multiple or ambiguous hashtags**

- *drinklocal*, *drunktwitter*

### Geographical Identification of the Prevalence of Alcohol-Related Hashtags

Next, we tested whether the knowledge of 24 significant alcohol hashtags could uncover information on alcohol-related outcomes in the United States. Alcohol-hashtagged tweets were filtered to 790,544 *geotagged* tweets containing longitude and latitude coordinate locations and mapped to metropolitan-micropolitan statistical areas (MMSAs), US county and county equivalents, and US states and Washington, DC. The total number of alcohol-hashtagged tweets in an area divided by the mean of the population estimates from the 2013-2018 American Community Survey yielded population-normalized alcohol-related hashtag prevalence.

We then tested the association between geographical prevalence of alcohol-related hashtags and alcohol outcomes. Spearman rho, a ranked nonparametric measure that is more robust to outliers than Pearson correlation, is used to report crude (nonadjusted) correlations [88]. Potential confounding variables previously studied in alcohol and social media use include race and sex distribution, median age, education, and income [37,89]. A linear regression analysis evaluated the relationship between the number of tweets per population and alcohol-related outcomes after including terms to control for confounding effects. Specific confounding variables from the 2013-2018 5-year American Community Survey report included *Percent Reporting White*, *Percent Reporting Black*, *Percent Reporting Hispanic*, *Median Income*, *Percent High School Education*, *Percent Bachelor's Degree Education*, and *Males/100 Females* [90]. All alcohol outcomes and confounding variables represented the most recent estimation of alcohol consumption and related behavior at the time of this study.

### Metropolitan-Micropolitan Statistical Areas

MMSAs are US Census Bureau designations of concentrated urban centers that may be the integrated areas of multiple cities and states (eg, the single *Washington-Arlington-Alexandria, DC-VA-MD-WV MSA* contains 3 US states and Washington, DC) [74]. The Behavioral Risk Factor Surveillance System publishes reports of survey responses at selected MMSAs for the following categories [91]:

- any alcohol consumption, defined as at least one alcoholic drink in the last 30 days;
- binge drinking behavior, defined as drinking >5 drinks in 1 event for men or >4 drinks in 1 event for women;
- heavy drinking, defined as drinking >1 drink per day for women or >2 drinks per day per man;

All yearly records from 2010-2019 for each MMSA were averaged to obtain a single number for outcome measurements.

### US County and County Equivalent

Primary US county outcomes were gathered from the University of Wisconsin Population Health Institute County Health Rankings and Roadmaps 2020 data, which included the estimates of excessive drinking, defined as the percentage reporting either binge or heavy drinking behavior as well as measurements of the percentage of motor vehicle fatalities that involved alcohol for the period between 2013 and 2018 [92]. In addition, data from the North American Industry Classification System provided by 2017 County Business Patterns (US Census) was used for the number of *Drinking Places (Alcohol Beverages)*; *Wineries*; *Breweries*; and *Beer*, *Wine*, *and Liquor stores* (North American Industry Classification System codes 722410, 312130, 312120, 445310, respectively) present in each county [93]. Counties were included if they contained at least one tweet and an average reported population >1000 between 2013 and 2018.

### US States and Washington DC

Twitter posts containing alcohol-related hashtags were aggregated by state and compared with the National Institute on Alcohol Abuse and Alcoholism's 2018 report, *Apparent Per Capita Alcohol Consumption: National, State, and Regional Trends*. This report predicts gallons of ethanol consumption based on alcohol sales and taxation data, separated for the consumption of wine, beer, or liquor products [94]. To determine which hashtags may be useful for detecting individual preferences of alcohol consumption, we calculated the correlation between the consumption of alcohol from different sources of alcoholic drinks (beer, wine, and liquor) and the prevalence of 19 beer, 5 wine, and 3 liquor-specific hashtags, as indicated in Textbox 1.

## Results

**Figure 3:**
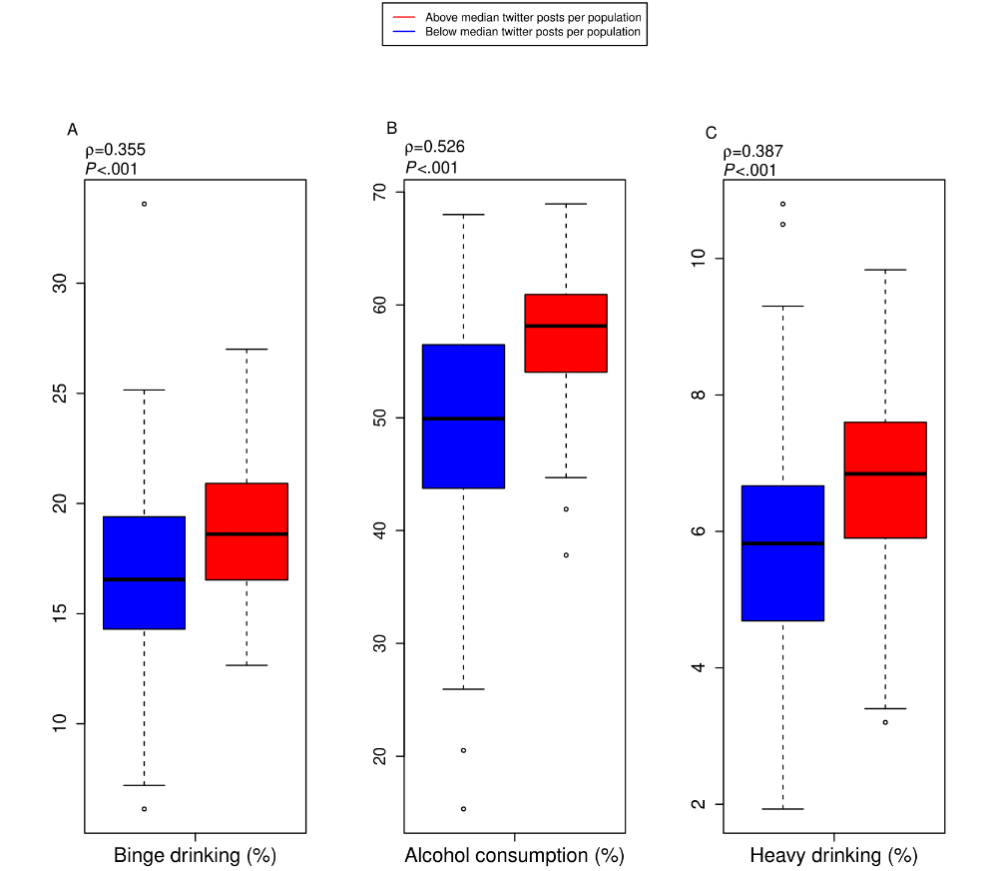
Metropolitan-micropolitan statistical area correlations for alcohol-hashtagged tweets and percent self-reported alcohol consumption. (A) Number of alcohol-hashtagged tweets and self-reported alcohol consumption within 30 days (N=179); (B) number of alcohol-hashtagged tweets and self-reported binge drinking within 30 days (N=179); (C) number of alcohol-hashtagged tweets and self-reported heavy drinking within 30 days (N=179).

Table 1 demonstrates the results from the analysis of alcohol-related hashtags and alcohol-related outcomes. The number of geotagged and alcohol-hashtagged tweets per population significantly correlated with many alcohol-related outcomes, including self-reported measures of individuals (1) reporting any alcohol consumption within 30 days (*P*<.001), (2) meeting the criteria for heavy drinking (*P*<.001), and (3) meeting the criteria for binge drinking (*P*<.001) at the MMSA level (Figure 3). However, the relationship between MMSA tweets and binge drinking level was not significant after adjusting for confounding effects (*P*=.37).

**Table 1:** Spearman correlation and linear regression results between the number of tweets per population and alcohol-related behavior and health indicators.

Outcome		Spearman correlation			Adjusted regression			Sample size, n
		ρ	*P* value	Coefficient β		*P* value		
**Metropolitan-micropolitan statistical area**								
	Alcohol consumption	0.526	<.001	1038		.01	179	
	Binge drinking	0.355	<.001	184.0		.37	179	
	Heavy drinking	0.387	<.001	244.8		.005	179	
**County and equivalent**								
	Excessive drinking	0.377	<.001	32.8		.03	2641	
	Percentage of alcohol motor vehicle fatality	0.063	.002	110.0		.21	2641	
	Drinking places (alcoholic beverages) per capita	−0.177	<.001	−2.18e–03		.23	1479	
	Breweries per capita	0.263	<.001	1.86e–03		<.001	334	
	Wineries per capita	0.130	.05	2.73e–02		<.001	228	
	Beer, wine, and liquor stores per capita	−0.043	.11	0.0039		<.001	1444	
**US states and Washington, DC, all hashtags**								
	Wine, gallons of ethanol per capita	0.756	<.001	74.11		<.001	51	
	Beer, gallons of ethanol per capita	−0.050	.73	9.911		.63	51	
	Liquor, gallons of ethanol per capita	0.320	.01	62.54		.03	51	
	All sources, gallons of ethanol per capita	0.437	<.001	146.6		<.001	51	
**US states and Washington, DC, hashtags stratified by alcohol category**								
	Wine, gallons of ethanol per capita (5 hashtags)	0.754	<.001	214.6		<.001	51	
	Beer, gallons of ethanol per capita (19 hashtags)	−0.001	.99	16.05		.63	51	
	Liquor, gallons of ethanol per capita (3 hashtags)	0.140	.33	338.0		.01	51	

**Figure 4:**
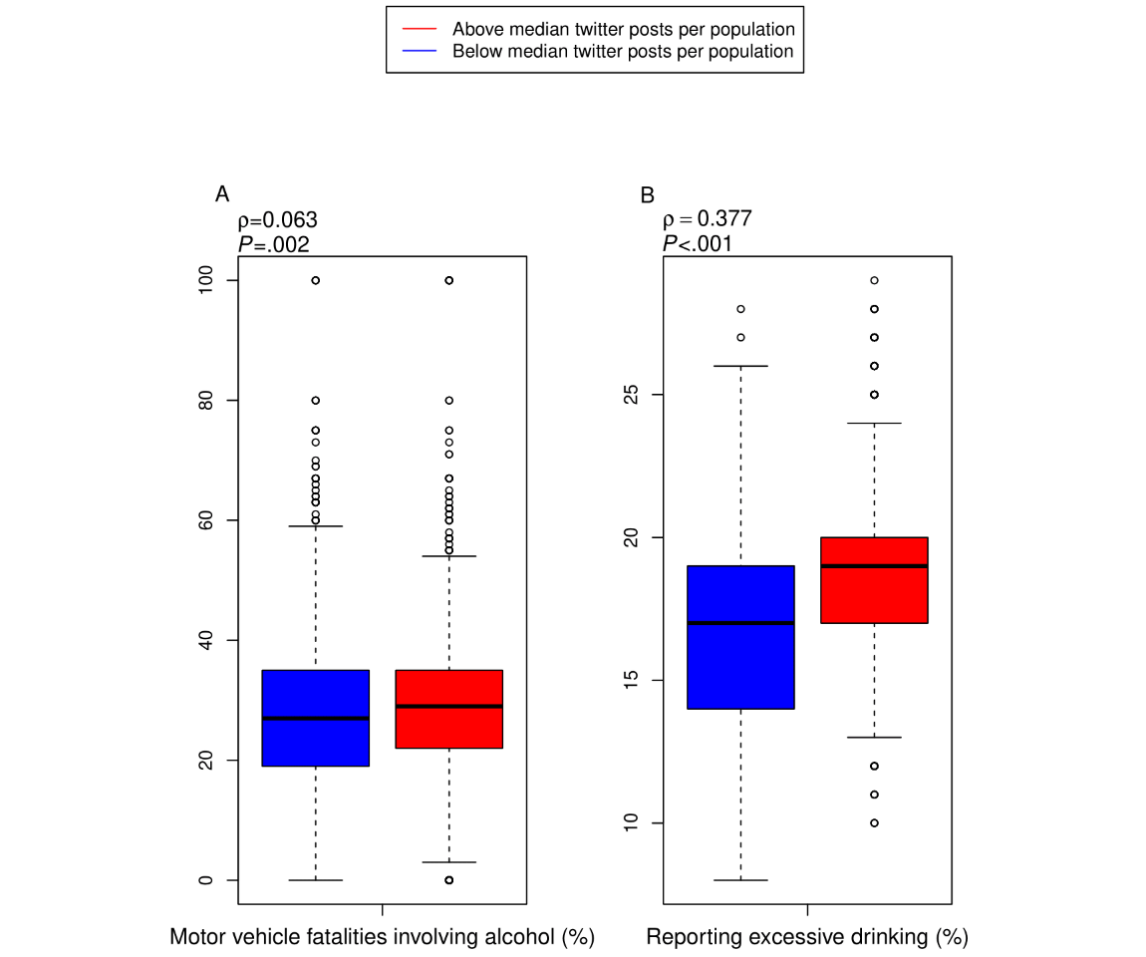
County- and county-equivalent–level correlations for alcohol-hashtagged tweets, alcohol motor vehicle fatalities, and self-reported excessive alcohol consumption. Counties are included if they contain at least one tweet and 1000 people (N=2641). (A) County-level correlations for alcohol-hashtagged tweets per population and percentage of motor vehicle fatalities involving alcohol. (B) County-level correlations for alcohol-hashtagged tweets per population and percent self-reporting excessive drinking (reporting either more than five alcoholic drinks on a single occasion for men or more than four alcoholic drinks on a single occasion for women, or more than two drinks per day for men or more than one drink per day for women).

There was a significant correlation between the percentage of motor vehicle deaths reported as involving alcohol (*P*<.001) and aggregated measures of excessive drinking behavior (*P*<.001) at the county level (Figure 4). However, the relationship between alcohol-related motor vehicle fatalities and county tweets per population was not significant after adjusting for confounding effects (*P*=.21).

**Figure 5:**
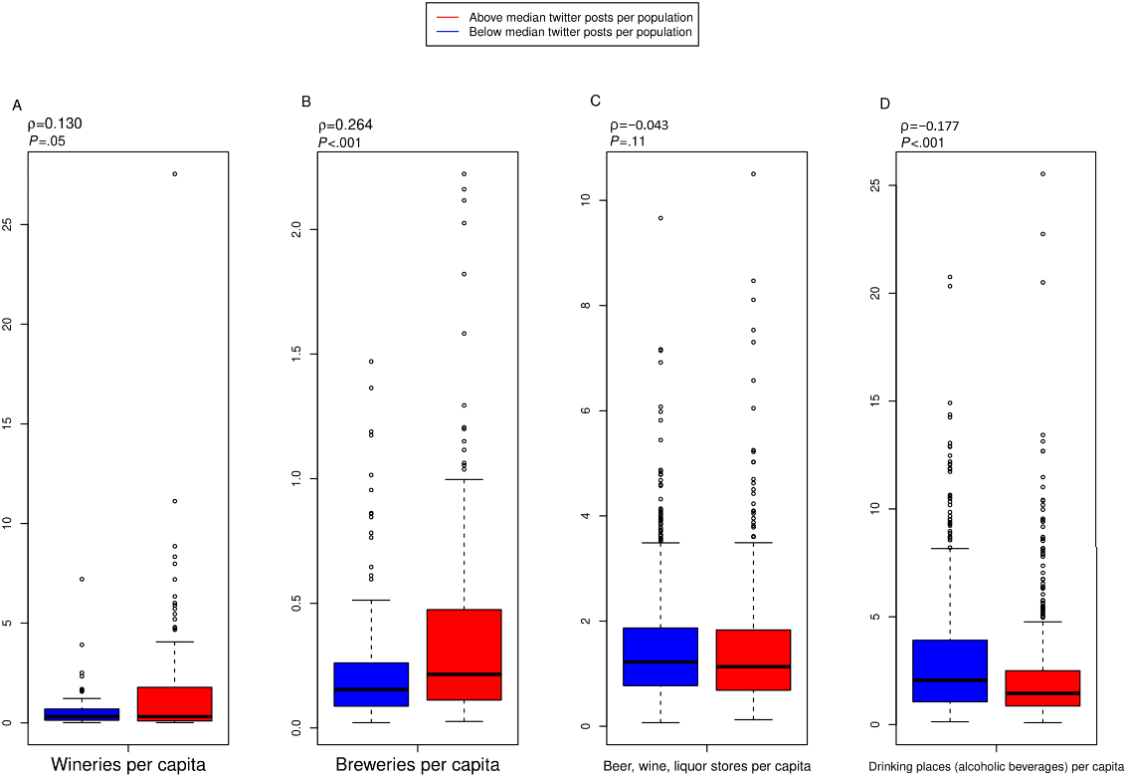
County- and county-equivalent–level correlations for alcohol-hashtagged tweets per person and alcohol-serving outlets, as reported by the North American Industry Classification System. Counties are included if they have at least one (1) tweet in our data set, one (1) alcohol outlet, and contain a population of 1000. (A) Wineries per 10,000 people (n=228 counties); (B) breweries per 10,000 people (n=334); (C) liquor stores per 10,000 people (n=1444); (D) drinking places (alcoholic beverages) per 10,000 people (n=1479).

There was a significant correlation between the number of alcohol-hashtagged tweets and wineries (*P=*.05), breweries (*P*<.001), and drinking places (alcoholic beverages; *P*<.001) but not beer, wine, and liquor stores (*P*=.11) per capita at the county level (Figure 5). However, after adjusting for confounding effects, there was a significant association between alcohol-related tweets per population and beer, wine, and liquor stores (*P*<.001) but not drinking places (*P*=.23) per capita.

**Figure 6:**
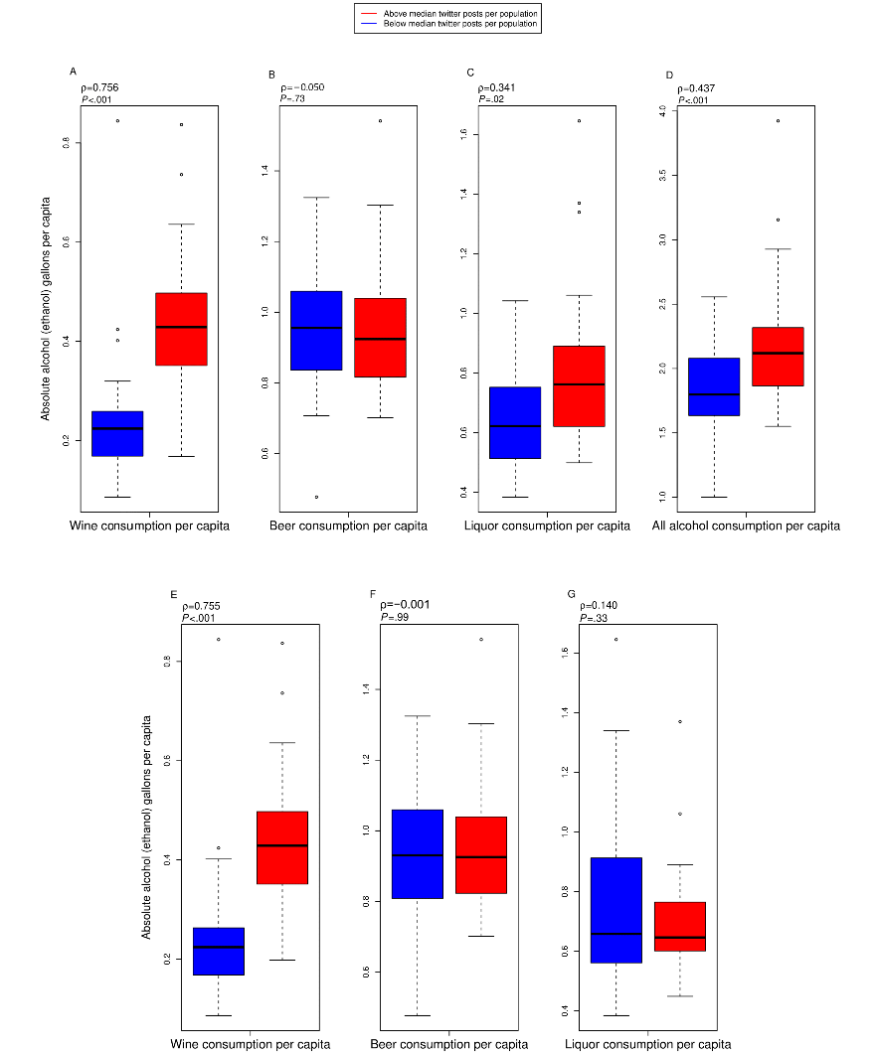
Gallons of ethanol consumed for US states and alcohol-hashtagged tweets. Population normalization was performed using the average reported population between 2010 and 2018, as reported by the National Institute of Alcohol Abuse and Alcoholism. Top, all 24 hashtags used; gallons of ethanol consumed from (A) wine; (B) beer; (C) liquor; and (D) all alcohol sources and Twitter posts per population. Bottom, only specific hashtags, gallons of ethanol from wine, beer, and liquor, and Twitter posts per population from specific hashtags: (E) wine (5 wine hashtags); (F) beer (14 beer hashtags); (G) liquor (3 liquor hashtags).

There was a significant correlation between the prevalence of alcohol-hashtagged tweets and gallons of wine (*P*<.001), liquor (*P*=.01), and overall gallons of consumption (*P*<.001) at the state level (Figure 6). However, the association with all alcohol-hashtagged tweets and gallons of beer consumed was not significant (*P*=.63).

The prevalence of five wine hashtags had a significant association with wine consumption at the state level (*P*<.001), but 3 liquor hashtags and 19 beer hashtags did not have a significant relationship with liquor (*P*=.33) and beer (*P*=.99) consumption at the state level. However, there was a significant relationship between gallons of liquor consumed and the prevalence of 3 liquor hashtags (*P=*.01) after controlling for confounding effects (Figure 6).

## Discussion

### Principal Findings

We demonstrated that information from alcohol-related subreddits could identify alcohol-related hashtags that correlated with multiple alcohol-related outcomes. The prevalence of geotagged tweets containing these significantly alcohol-related hashtags correlates with alcohol-related behaviors and alcohol outlet density, which are associated with adverse outcomes, including deaths from motor vehicle crashes and excessive drinking [95-97]. This approach has distinct benefits for studying alcohol-related outcomes. Compared with other approaches to detect alcohol consumption on Twitter, the pipeline presented here is trained using relatively few interpretable input and output parameters, specifically, 18 subreddits and 24 hashtags. The NLP pipeline identified language associated with alcohol abuse on Twitter without manual annotation or predefined keywords and with only the knowledge of relevant subreddits. These results indicate that alcohol-related language, when defined by inclusion into the themed communities of subreddits or containing alcohol-related hashtags, can be used to understand population-level behavior in multiple geographic areas with different population granularity.

Qualitatively, the model presented here detects a wide variety of different alcohol-related hashtags, including slang. Tweets containing these hashtags capture a large amount of information regarding alcohol consumption behavior at a broad population level. The set of hashtags resulting from this study is useful for future alcohol-related research on Twitter and identifying relevant hashtags or community forum labels on other platforms.

A notable benefit of using hashtags and subreddits as platform-specific labels for studying alcohol-related outcomes is interpretability. Although deep learning models excel at understanding massive quantities of data, many NLP and deep learning models rely on complex feature representations to classify or characterize text. However, this approach is not easily interpretable [65,98]. We have demonstrated that by treating subreddits and hashtags as learnable labels, it is possible to directly use hashtags as interpretable features in our NLP pipeline for social media data to understand alcoholic beverage preferences while still learning from a large amount of data.

Consumption of different alcohol types is associated with a variety of both beneficial and detrimental outcomes [99-101]. For example, wine consumption is associated with protection from cardiovascular diseases; however, the cause of this effect may be dietary factors and other lifestyle choices [99,102]. Other studies have associated preferential beer and liquor consumption with adverse outcomes, such as dangerous drinking and other risky behaviors [101,103,104]. Notably, although many studies have examined overall alcohol mentions on Twitter, few models created have been explicitly examined in terms of differences in the types of alcoholic beverages mentioned.

Our study indicates that information capturing consumption of wine and liquor is directly observable using social media data, as shown by the significant associations between the prevalence of 5 wine-related hashtags and the amount of wine consumed, as well as the number of posts containing at least 1 of the 3 liquor-related hashtags and liquor consumed. However, there was no significant relationship between beer consumption per capita and the number of alcohol-hashtagged Twitter posts in an area. The results here indicate that our model can detect certain types of alcohol consumption behavior (wine and liquor consumption) on Twitter using the interpretability of hashtags but not others (beer consumption). It remains unclear whether our results indicate a bias in our model’s methodological choice (eg, use of hashtags or training procedure) or a difference in social media populations that prefer different alcohol beverages. The difference in correlations might be because of several variables, including the existence of confounding factors related to the prevalence of social media use in the underlying populations that preferentially consume beer over other alcohol types, differences in perceived acceptance of beer consumption behavior, or because of other factors that may confound alcohol and social media posting [89]. Alcohol preference is an example of having interpretable hashtag representations for a given model that may help identify behavioral differences associated with an outcome of interest. This evidence suggests that similar models trained on Twitter may detect alcohol wine and liquor consumption but not beer.

### Comparison With Previous Works

Many previous studies have used hashtags as target labels. However, they mostly rely on a predefined set of hashtags that may not be data-driven or require extensive expert annotation instead of taking advantage of topic-specific sources and social media content on other public social media platforms [40,105-107]. Notably, using a predefined number of hashtags could be biased and too narrow for capturing relevant information, potentially missing informative hashtags for exposure or outcome. Traditional keyword approaches may fail to capture various pieces of information from slang and novel hashtags from platform-specific languages as they are created and popularized. In addition, keyword databases may not exist for all outcomes of interest. Semantic similarity measures, such as Word2vec, may identify hashtags with similar contexts; however, integrating vector representations may lose valuable information relative to training over individual samples, and prediction probabilities or certainty are not readily observable. In contrast, this study indicates that a model trained on a large set of data relevant to the behavior of interest and its application to an unlabeled data set from a different platform can identify data-driven hashtags related to that behavior. This ability to learn hashtags from data is critical, as new hashtags are created every day and may differ substantially between platforms.

Many social media platforms are directly searchable using hashtags, allowing the ability to gather many highly specific posts instead of gathering a large number of nonspecific posts to identify relevant hashtags, keywords, or alcohol content based on available prevalence data. Although the latter approach has shown success in studying alcohol-related behavior previously, methods to extend the analysis to alternative but potentially related outcomes, platforms, or different geographical designations remain unclear [39,40,108]. The generalization of such models necessitates the creation of individual models for each particular outcome and geographic area. Finally, these models may fail to take advantage of extensive research on themed communities to understand alcohol use and other outcomes of interest [30,39,40,76,106,109,110]. In contrast, the method outlined here may help create more efficient public health interventions to analyze the alcohol consumption behavior for a given geographic area of interest, such as a city or hospital catchment area. This approach is particularly useful when the relevant language is dynamic or contains area-specific slang, making previously established dictionary-based methods incomplete or impractical. In particular, our proposed methodology for identifying hashtags using a previously trained deep learning model can be useful for detecting alcohol consumption behavior on various social media platforms.

### Limitations and Future Work

There are some limitations to the model and approach proposed in this study. Other health-related behaviors that are not discussed frequently on social media may be more difficult to ascertain and translate between platforms using hashtags. Any model trained on a limited number of social media platforms may be confounded by differences in user preferences, such as age or socioeconomic status. Furthermore, this method relies on identifying known or previously studied subreddits, which may not be suitable for outcomes without known relevant subreddits. Furthermore, we did not compare our models’ performance with the BERT large model or other deep learning alternatives, which can have a different performance for our task. In addition, including additional social media data in model development and using domain knowledge from ontologies, controlled vocabularies, lexicons, and relevant rules and regular expressions can further improve the presented results.

As future work, we plan to extend this study to other mental and behavioral health topics, such as depression and substance use, and other social media platforms that use hashtags, such as Facebook and Instagram. Deep learning has previously been used to combine the analysis of images and texts from social media users [38]. In our future work, we will expand the presented architecture to include other data modalities, such as images and videos, to increase screening capabilities.

### Conclusions

These results indicate that using alcohol-related subreddits as learnable labels to train a BERT neural network can capture interpretable, alcohol-related language on Twitter. Our study suggests a significant correlation between the prevalence of alcohol-related geotagged Twitter hashtags and alcohol-related behaviors as measured by self-reported alcohol consumption, alcohol preferences, and alcohol outlet prevalence. This method has the unique advantages of previous methods, including allowing examination at the MMSA, US county, and US state level for different alcohol-related outcomes. These results suggest that using previously studied hashtags and subreddits as learnable targets in a machine learning framework could expand public health outreach efforts and epidemiology research, particularly for monitoring behavior related to alcohol consumption.

## Abbreviations

API: application programming interface
BERT: Bidirectional Encoder Representations From Transformers
MMSA: metropolitan-micropolitan statistical area
NLP: natural language processing
